# Community recruitment of underrepresented populations to a preclinical AD trial using novel partnerships with nursing organizations: Lessons and outcomes

**DOI:** 10.1002/alz.090971

**Published:** 2025-01-09

**Authors:** Christian R Salazar, Melanie B Tallakson, Maria Corona, Edwin Duran, Eunji Russ, Dan Hoang, Romina A Romero, David L Sultzer, Joshua D Grill, Hye‐Won Shin

**Affiliations:** ^1^ Institute for Memory Impairments and Neurological Disorders, University of California, Irvine, Irvine, CA USA; ^2^ University of California, Irvine, Irvine, CA USA; ^3^ School of Medicine, University of California, Irvine, Irvine, CA USA; ^4^ The UC Irvine Institute for Memory Impairments and Neurological Disorders (UCI MIND), Irvine, CA USA; ^5^ Somang Society, Cypress, CA USA

## Abstract

**Background:**

Alzheimer’s disease (AD) disproportionately affects minoritized populations, who are consistently underrepresented in clinical trials. We aimed to recruit underrepresented adults aged 55‐80 to an ongoing preclinical AD trial.

**Method:**

A community‐based strategy was used to recruit Filipino, Hispanic/Latino, and Korean adults into the AHEAD study, a preclinical AD trial testing the anti‐amyloid treatment lecanemab, at the University of California, Irvine. We forged partnerships with nursing and community‐based organizations to facilitate educational and engagement events tailored to each community’s cultural and linguistic needs. The educational content provided a comprehensive overview of the AHEAD Study, its objectives, methodology, and eligibility criteria, alongside an in‐depth explanation of dementia, AD, its risk factors, and lifestyle modification strategies for risk reduction. During pre‐screening, potential participants underwent a brief in‐person or telephone review of qualifications and were referred to the study site for formal screening.

**Result:**

Through 21 community education and engagement events we reached 654 individuals, leading to the pre‐screening of 71 individuals, of whom 11.2% were Hispanic/Latino adults, 21.1% were Filipino adults, and 67.6% were Korean adults. Among Hispanic/Latino adults who pre‐screened, 50% were deemed ineligible primarily due to age requirements. Approximately 47% of the Filipino adults who pre‐screened did not proceed to in‐person screening, with the primary reasons being withdrawn interest and loss to follow‐up. Of the Korean adults who pre‐screened, 69% were found ineligible, predominantly due to challenges with English fluency and loss to follow‐up. A total of 25 participants, representing 3.8% of those reached, were successfully enrolled in the AHEAD Study. Of these, 2 (8%) were Hispanic/Latino, 8 (32%) were Filipino, and 15 (60%) were Korean adults. The success of the study depended upon partnerships with nursing and community‐based organizations, leveraging existing trust and rapport. Continuous communication and early nurse leader involvement as well as participant feedback in refining educational content for cultural relevance were key.

**Conclusion:**

Community‐based recruitment strategies that include trusted local organizations and culturally sensitive approaches were effective in engaging underrepresented groups in interventional AD research.

